# Corneal, Scleral, Choroidal, and Foveal Thickness in Patients with Rheumatoid Arthritis

**DOI:** 10.4274/tjo.58712

**Published:** 2017-12-25

**Authors:** Onur Gökmen, Nilüfer Yeşilırmak, Ahmet Akman, Sirel Gür Güngör, Ahmet Eftal Yücel, Hilmi Yeşil, Fatih Yıldız, Adam Sise, Vasilios Diakonis

**Affiliations:** 1 University of Health Sciences, Van Training and Research Hospital, Department of Ophthalmology, Van, Turkey; 2 University of Health Sciences, Ankara Training and Research Hospital, Department of Ophthalmology, Ankara, Turkey; 3 Başkent University Faculty of Medicine, Department of Ophthalmology, Ankara, Turkey; 4 Başkent University Faculty of Medicine, Department of Rheumatology, Ankara, Turkey; 5 University of Health Sciences, Van Training and Research Hospital, Department of Rheumatology, Van, Turkey; 6 Bascom Palmer Eye Institute, Miami, USA; Miller School of Medicine, Florida, USA

**Keywords:** Rheumatoid arthritis, scleral thickness, corneal thickness, choroidal-retinal thickness, optical coherence tomography

## Abstract

**Objectives:**

To investigate corneal, scleral, choroidal, and foveal thicknesses in female patients with rheumatoid arthritis (RA) and compare them with healthy subjects.

**Materials and Methods:**

This prospective study included consecutive female patients diagnosed with RA and healthy subjects. Corneal, scleral, choroidal, and retinal (foveal) thicknesses were obtained by using optical coherence tomography and a comparison was performed between groups for all outcome measures.

**Results:**

Thirty-six eyes of 36 female patients diagnosed with RA (group 1) and 36 eyes of 36 healthy female volunteers (group 2) were included. Mean corneal, scleral, choroidal thicknesses and retinal thickness at the fovea of group 1 were 543.3±33.7 µm, 343.7±42.2 µm, 214.6±50, and 213.5±18.9 µm, respectively; in group 2, these values were 549.9±29.6 μm, 420.9±42.4 μm, 206.4±41.9 μm, and 222±15.5 μm, respectively. The comparison between group 1 and 2 with respect to corneal, choroidal, and foveal thicknesses did not reveal statistical significant differences (p>0.05). On the contrary, there was a statistically significant difference with respect to scleral thickness between the groups, with the RA patients demonstrating a thinner scleral layer (p<0.001).

**Conclusion:**

Female patients with RA seem to demonstrate statistically significant scleral thinning when compared with healthy subjects, while there was no difference concerning corneal, choroidal, and foveal thickness.

## INTRODUCTION

Rheumatoid arthritis (RA) is a systemic inflammatory disease which primarily affects the joints and may also manifest with extra-articular symptoms.^1^ Ocular manifestations can be found in 25% of patients with RA, and include keratoconjunctivitis sicca, peripheral corneal ulceration, keratitis, episcleritis, scleritis, choroiditis, and retinal detachments.^2,3^ Patients with RA not treated with effective immunosuppressive therapy may develop peripheral ulcerative keratitis, necrotizing scleritis, corneal and scleral perforations, which may lead to visual function decrease and thereby seriously reduce patients’ quality of life.^4^

Scleral inflammation caused by RA can manifest as mild episcleritis or full-thickness scleritis, which can rarely result in scleral melting.^5^ Scleral thickness has been measured in previous studies with ultrasound biomicroscopy as well as with anterior segment optical coherence tomography (OCT) in glaucomatous patients. In this study we assessed scleral thickness using OCT in patients with RA and compared them with healthy volunteers. Furthermore, we also utilized enhanced depth imaging (EDI)-OCT, which is a newer technique allowing cross-sectional imaging of the retina and choroid. EDI-OCT and anterior segment OCT have been used to evaluate retinal and choroidal thickness in many ophthalmic diseases; however, there is a lack of studies investigating patients with RA.^6,7^ The current study prospectively investigated corneal, scleral, choroidal, and foveal thicknesses in female patients with RA and compared the outcomes with healthy female subjects.

## MATERIALS AND METHODS

### Patient Population

This prospective study included female patients that were diagnosed with RA in accordance with the 2010 RA classification of the American Rheumatism Association in the Rheumatology Department of Başkent University in Ankara, Turkey between June and December 2014. Two groups of subjects were assessed in this study; group 1 included eyes of female patients diagnosed with RA and group 2 consisted of eyes of healthy female subjects.

The study was approved by the Başkent University Institutional Review Board and Ethics Committee (KA 14/26). The research adhered to the tenets of the Declaration of Helsinki, and detailed written informed consent was obtained before each individual’s participation in the study.

### Inclusion and Exclusion Criteria

Female patients over the age of 45 years with positive rheumatoid factor and were diagnosed with RA in accordance with the 2010 RA classification of the American Rheumatism Association were included in the study. In order to avoid refractive error magnitude influencing the main outcome measures (corneal, choroidal, and scleral thickness), we included patients with spherical refractive error between +2 and -2 diopters. Exclusion criteria included any corneal or lenticular opacity other than mild cataract, history of trauma or surgery that involved the conjunctiva or sclera, and history of any other connective tissue disease. Patients with active or resolved scleritis, episcleritis, keratitis, or uveitis or any history of these were excluded.

### Patient Assessment

**Figure 1:**
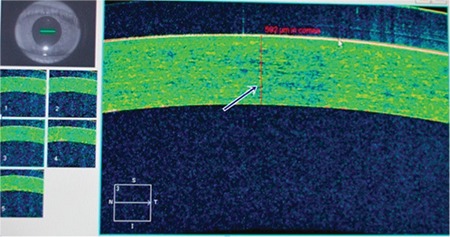
Corneal thickness measurements were obtained during straight gaze position with optical coherence tomography

**Figure 2:**
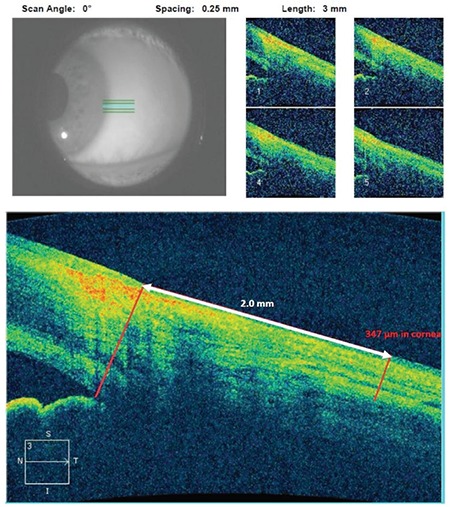
Scleral thickness measurements were obtained during 45 degree temporal gaze position with optical coherence tomography

The participants underwent a complete ophthalmological examination which included slit-lamp clinical evaluation of the central cornea, perilimbal sclera, central retina, and choroid. Corneal and scleral thickness was measured using the Cirrus HD-OCT (Carl Zeiss Meditec, Dublin, CA, USA); choroidal and foveal thicknesses were measured using the Heidelberg Spectralis OCT (Heidelberg Engineering, Heidelberg, Germany). Corneal thickness measurements were obtained with the eyes in primary gaze position, while scleral thickness measurements were obtained using a 45° temporal gaze to measure the sclera 2 mm nasal to the corneal limbus (Figures 1 and 2).

**Figure 3:**
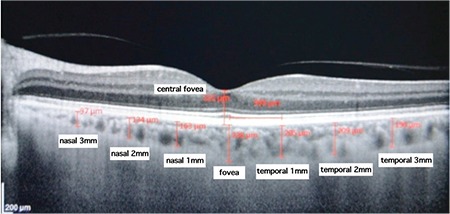
Measurement of choroidal thickness with enhanced depth imaging-optical coherence tomography from the outer edge of the retinal pigment epithelium (RPE) to the choroid/scleral boundary at the fovea and at 6 more points located at 1 mm nasal to the fovea, 2 mm nasal to the fovea, 3 mm nasal to the fovea, 1 mm temporal to the fovea, 2 mm temporal to the fovea, and 3 mm temporal to the fovea. Retinal thickness was measured from the internal limiting membrane to the RPE at the fovea

Choroidal thickness was measured perpendicularly from the outer edge of the retinal pigment epithelium (RPE) to the choroid/scleral boundary at the fovea and at 6 more points located at 1000 µm nasal to the fovea, 2000 µm nasal to the fovea, 3000 µm nasal to the fovea, 1000 µm temporal to the fovea, 2000 µm temporal to the fovea, and 3000 µm temporal to the fovea. Retinal thickness was also measured manually from the internal limiting membrane to the RPE at the fovea (Figure 3) using the caliper provided by OCT software. All measurements were taken by two independent blind researchers (O.G. and S.G.G.) and were averaged for statistical analysis. Participants were asked not to consume drinks containing caffeine and/or eat chocolate three hours prior to OCT assessment to avoid possible effects on choroidal thickness.

### Statistical Analysis

Statistical analysis was performed using SPSS version 20.0 (Statistical Package for the Social Sciences) software. For each variable, normality was checked by Kolmogorov-Smirnov and Shapiro-Wilk tests. Mann-Whitney U test was used to evaluate statistical differences in corneal, scleral, retinal, and choroidal thicknesses between groups 1 and 2. Correlation analysis between disease duration and measurements of scleral and corneal thicknesses were performed by using Spearman’s Rho test. Values of p<0.05 were considered statistically significant.

## RESULTS

This prospective study included a total of 72 eyes of 72 female patients. Group 1 comprised 36 eyes of 36 female RA patients aged 56.12±9 years (range, 45-69 years); group two comprised 36 eyes of 36 healthy females aged 58.13±8 (range, 45-68 years). Therefore, a total of 86 measurements were done; however, 14 measurements were excluded due to poor image quality. All the RA patients were under immunosuppressive therapy and were followed regularly by their rheumatologists. Disease duration after initial diagnosis in the study group (group 1) was 8.6±1.2 years (median 5.5, range 2-40 years). The groups showed no significant difference in age (p>0.05).

Mean corneal thickness was 543.3±33.7 µm (range, 444-612 µm) in group 1, and 549.9±29.6 µm (range, 496-596 µm) in group 2; there was no statistical difference between the groups (p>0.05). Mean scleral thickness was 343±42.2 µm (range, 268-596 µm) in group 1 and 420.9±42.4 µm (range, 354-544 µm) in group 2. The difference between the groups was statistically significant (p<0.01). Mean retinal thickness as measured from the fovea to RPE was 213±18.9 µm (range, 153-249 µm) in group 1 and 222±15.5 µm (range, 180-256 µm) in group 2; the difference was not statistically significant (p>0.05) (Table 1). Mean choroidal thickness was averaged from seven points at 1000 μm intervals from temporal to nasal choroid across the fovea. The differences between the two groups were not statistically significant (p>0.05) (Table 2). No correlation was found between disease duration and corneal thickness or scleral thickness (p=0.316).

## DISCUSSION

RA is usually associated with extra-articular findings. Turesson et al.^8^ evaluated 609 RA patients from 1955 to 1994 and showed that 247 patients (41%) had at least one extra-articular finding. The most frequent ocular manifestation of RA is keratoconjunctivitis sicca.^8^ Other reported ocular complications of RA are episcleritis, scleritis, retinal vasculitis, peripheral ulcerative keratitis, and interstitial keratitis.^9^ Cytokines such as interleukin 1 (IL-1), IL-6, and tumor necrosis factor alpha (TNF-α) are believed to play a major role in the development of extra-articular findings in RA. The efficacy of anti-TNF-α agents like infliximab further support this hypothesis.^10^ An imbalance of these pro- and anti-inflammatory cytokines creates a microenvironment that supports the breakdown of collagen in RA. This can manifest as keratitis starting from the perilimbal cornea and spreading toward the central cornea, causing corneal melting and perforations.^11,12^

The mechanisms of action of RA suggest that manifestations in different ocular tissues and macrostructural tissue changes (pachymetric alterations, etc.) could occur in patients with RA. In our study we assessed corneal, scleral, choroidal, and foveal thickness in order to identify possible implications of RA on macrostructural tissue alterations with respect to thickness. According to the literature, most of the studies have measured central corneal thickness in RA patients using confocal microscopy, pachymetry, Scheimpflug imaging systems, and ocular response analyzers. However, none of them utilized OCT.^13,14,15,16^ Therefore, we aimed to measure central corneal thickness using anterior segment-OCT in RA patients and we did not detect a statistical significant difference between patients with RA and healthy subjects with respect to central corneal thickness. This finding may suggest that patients actively managed with immunomodulatory agents (like the RA patients in this study) do not do demonstrate corneal thinning.

The choroidal layer is the most vascularized layer in the eye, so it can play a role in many ophthalmologic diseases. Newly developed OCT applications (EDI-OCT) allowing cross-sectional imaging of the choroid, and several studies have demonstrated that choroidal thickness changes in ocular diseases such as age-related macular degeneration, high myopia, chorioretinal atrophies, Vogt-Koyanagi-Harada disease, Behçet’s disease, sarcoidosis uveitis, and polypoidal choroidal vasculopathy.^6,17,18^ Despite improvements in software analysis, studies have also shown that manual retinal and choroidal thickness measurements are still superior to automated measurements.^19,20^ In the current study we measured choroidal and retinal thicknesses of RA patients manually with the caliper provided by the Spectralis OCT software. According to our results, there was no statistically significant difference with respect to choroidal and retinal thickness when compared to healthy subjects. As our patients were all under active immunomodulatory treatment, this may have prevented choroidal tissue alterations; possible changes in choroidal thickness may be evident in patients with uncontrolled RA. Nevertheless, choroidal involvement is rare in RA; it is usually a finding after inflammation due to posterior scleritis, which in turn is also rare. Therefore, immunosuppressive treatment may not be the reason for this, as retinal changes are very rare in RA. Given that the current study has a small sample size, the absence of structural changes of the retina may not be indicative. Further studies including patients with uncontrolled RA are needed to investigate the effect of RA on the choroid. 

Scleritis in RA has a reported prevalence of 0.63-0.67%. Furthermore, scleral thinning and perforation due to inflammatory vasculitis or scleromalacia perforans rarely occurs in patients with RA.^21^ While scleritis may lead to scleral thinning and melting, the pathogenesis of scleritis in RA is still unknown.^22^ In our study, although none of our RA patients had active scleritis, inflammatory vasculitis, or scleromalacia perforans, they demonstrated a significant decrease in scleral thickness when compared to the healthy subjects (p<0.001). The etiology of this thinning is unclear but may be related to subclinical immune complex deposition and destruction of scleral tissues despite appropriate immunosuppressive therapy. In addition, we evaluated scleral thickness as correlated to disease duration and found no correlation between them. However, the disease progression rate can be quite variable, and the small size of our study may have affected this correlation.

### Study Limitations

A limitation of the current study is the small number of eyes included. Furthermore, there is a selection bias with respect to the gender included in the study; we only included female patients as the number of male patients was very small. Our inability to include more male patients can be attributed to the 3-fold higher incidence of RA in the female population when compared to males.^23^ Another limitation of the current study is the lack of a positive control (a group of subjects with diseases other than RA that manifest with scleritis). This would add to the current study and provide further scientific validation of the outcomes described herein. We only included a normal (healthy) group of female patients that served as controls, as we did not locate a sufficient number of subjects with diseases other than RA that manifest with scleritis. Finally, the images acquired by the Cirrus HD-OCT are not of high resolution and quality due to the capabilities of the current imaging platform; nevertheless, pachymetric measurements should not be influenced by this limitation.^24,25^

## CONCLUSION

This study shows that scleral thinning occurs in female patients with RA under active immunomodulatory treatment and no active scleritis, inflammatory vasculitis, or scleromalacia. This could be caused by a chronic inflammatory process, subclinical immune complex deposition, or direct destruction of scleral tissues by autoimmune reaction. Patients with RA could be followed for possible scleral thinning and perforations as there seems to be a tendency towards thinning despite appropriate immunomodulatory control. Corneal, retinal, and choroidal thicknesses, however, should be normal in properly treated patients.

## Figures and Tables

**Table 1:**
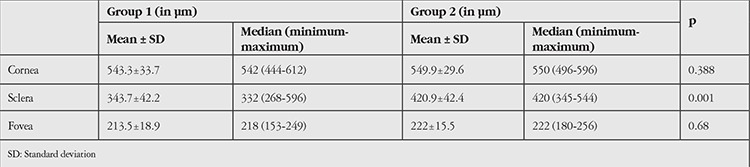
The mean and median corneal, scleral and foveal thicknesses of patients and controls

**Table 2:**
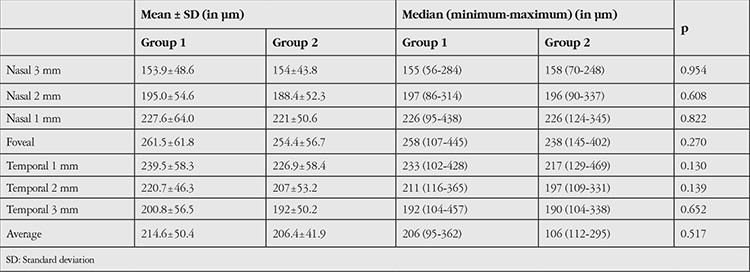
The mean and median choroidal thicknesses of patients and controls at 7 points
